# Supplementary material for: Identifying engagement strategies for Hispanic youth with anxiety: A youth-centered, Design-Thinking approach
Source: PLoS One. 2025 Feb 10;20(2):e0317142. doi: 10.1371/journal.pone.0317142 (PMC11809788; doi:10.1371/journal.pone.0317142)
Supplement: S1 Table — From key informant interviews. (DOCX) [file pone.0317142.s001.docx]

# Supplementing Information

# **SI Table.** **Codebook from teen interviews.**

| ***Theme*** | ***Sub -theme(s)*** | ***Description*** |
| --- | --- | --- |
| Personal factors | | |
| Causes of Anxiety | **What teens believe to be the most common causes of anxiety.** | |
|  | School | A source of anxiety for teens that includes academics and relationships with peers, teachers and staff at school. |
|  | Social Situations | Anxiety caused by being around a lot of people and in large social situations, especially at school. |
|  | Social Apps | Social media apps identified as a source of anxiety. |
| Barriers to Seeking Help | **The psycho-social and structural barriers that impact a student's decision to seek help.** | |
|  | Lack of Trust | Prefer not talking with someone about how they are feeling because they don't know that person well enough to trust them. |
|  | Fear of Judgment | Fear of being judged or misunderstood so teen is not comfortable sharing how they feel. |
|  | Attitude Towards Therapists | Teens’ negative perceptions of therapy and therapists prevent them from seeking help. |
| **Behavioral factors** | | |
| Coping Strategies | **Coping strategies that teens use to help manage their feelings of anxiety.** | |
|  | Physical activity | Physical activity and exercise as a tool to help manage anxious feels. |
|  | Journaling | The action of writing down or recording down how they are feeling. |
|  | Social Media | Social media used as a distraction from anxious feelings or as a source of advice on how to deal with anxiety. |
|  | Music | Listening to music and creating playlists to calm them down or distract them. |
| **Environmental factors** | | |
| **School Resources** | **Resources available at school to help teens when they are feeling anxious.** | |
|  | Wellness Rooms | Rooms at school where teens can go take a "brain break" when feeling anxious during the school day. |
|  | School Counselor | A school counselor assigned to a teen primarily to assist with academics. |
|  | Teacher/Mentor | A trusted adult at school that the teen goes to for support. |
|  | School Therapist | A licensed therapist available at school. |
| **Sources of Support** | **The people that teens go to for help and support when feeling anxious.** | |
|  | Trusted Adult | An adult who is not their parent that the teen feels like they can trust and share their feelings with (school administrator, teacher, mentor, relative). |
|  | Friends | Teens said they turn to friends for support. |
|  | Family Members | Teens said they would turn to a family member for support. |
|  | Self | Teens would rather keep it to themselves than talk to someone else. |
|  | Therapy | Available but not mentioned as a source of support. |
| **Program ideas** | **Teen ideas of what they and their peers would want in a program or product designed to help feelings of anxiety.** | |
|  | Education & Coping Skills | Education about mental health and effective coping strategies. |
|  | Interactive Games & Activities | Fun interactive games and activities that teach them new things. |
|  | Access to Trusted Adult | Ability to contact a trusted adult for support and advice. |
|  | Things to Consider | Extra advice about how to engage students with a new program. |
