# Supplementary material for: Identifying engagement strategies for Hispanic youth with anxiety: A youth-centered, Design-Thinking approach
Source: PLoS One. 2025 Feb 10;20(2):e0317142. doi: 10.1371/journal.pone.0317142 (PMC11809788; doi:10.1371/journal.pone.0317142)
Supplement: Attachment — Submitted filename: Response to Reviewers.docx [file pone.0317142.s002.docx]

**Response to Editor:**

**We note that Figure 2 includes an image of a participant in the study.**

Regarding the concerns about the photo in Figure 2, this does not include an image of a study participant. It is a publicly available stock photo used to help students connect with the persona.

**Reviewer #1: 1. Main Claims:
This work identifies that Hispanic youth with anxiety challenges want culturally relevant technology-based programs providing easily accessible educational information and coping strategies that are engaging and facilitate the support of a trusted adult.

It is worth noting that the goal of the research is stated as follows: “to better understand the barriers and facilitators to youth engagement with mental health services and to identify the important criteria to consider for the design of and engaging intervention that would encourage Hispanic youth to seek help for anxiety and keep them engaged with treatment.” The assumption is that they mean Hispanic youth in the US, since this would be different if it were a study conducted in a Central or South American society, for instance. For clarity, that caveat should be included in the statement of the goal of the research.**

**This goal is stated as about engagement in treatment, not treatment itself. Yet the title of the paper states that it is about designing a treatment. Please align the title with the actual outcome of the research.**

RESPONSE:

We agree that the title and goal statement could better reflect the focus on understanding engagement facilitators and barriers rather than delivering a treatment. We revised the title to align with our aims more accurately and focus on youth engagement. The new title is: Identifying Engagement Strategies for Hispanic Youth with Anxiety: A Youth-Centered, Design-Thinking Approach. Additionally, we clarified that this study targets Hispanic youth in the U.S in the goal statement (p. 4)

2**. Context with Previous Literature:
The discussion of the literature focuses on barriers to accessing treatment for Hispanic youth and low-income youth with anxiety in the US and the implications of no treatment for anxiety.

There is only one reference to the Design Thinking methodology in the Introduction (plus a second one in the methods section). The Introduction would benefit from more elaboration on this approach and the potential strengths and weaknesses of it. This is particularly important since the novelty of the study is described (in the Discussion) as having used this methodology.**

RESPONSE:

We provided additional context on the strengths of using Design Thinking as a human-centered approach that uses core concepts of empathy, co-creation and problem solving and the rationale for why we selected this particular methodology. P. 4- 5

**3. Data Analysis Support:
This study utilizes Design Thinking as it’s methodological foundation. That involves 5 stages – empathizing, defining, ideating, prototyping and testing. The authors note that they only conducted the first 3 of these: empathizing, defining and ideating. It would be clearer if the authors acknowledge up front that they are not using a full Design Thinking approach with the goal of creating solutions. Rather, they are using the first 3 phases of Design Thinking to better define the problem and identify domains of solutions.** RESPONSE: Thank you for acknowledging the importance of specifying our Design Thinking stages. We adjusted the Methods section to clarify that we used the first three phases of Design Thinking to better define the problem. We clarified that the goal was not to create a full intervention solution but rather to explore problem domains and potential criteria for youth-centered engagement in mental health support (p.5)

**The study is reported to have recruited low income participants. Yet there were no data presented to define what that means or verify that it pertained to each of the participants. I don’t believe that we should assume that all Hispanic youth in a particular school or location are low income, but that does depend on how the construct is defined and that assumption would need to be justified.**

RESPONSE: Although recruiting from low-income communities, data was not collected to verify participant SES so we removed low-income and focused on Hispanic youth.

**The first stage of the project involves key informant interviews about help-seeking behaviors. The section on Key informant interviews has a list of the questions asked, which is helpful. There is mention of a Fig 1 Social Cognition Theory. The first actual figure is Fig 2. This is followed by Figs 3 and 4 and then, finally Fig 1.**

RESPONSE: We reviewed the Figures. We deleted the reference to Fig 3 on line 255 to avoid confusion. The order of figures should be correct.

**The stated goals and methodological details need to influence the title of the paper. It is not the case that the paper describes the “design of an intervention for the treatment of anxiety”. Rather, it describes supports that can be useful to help engage Hispanic youth with anxiety. Treatment is more extensive and can be defined more specifically than the supports identified by the qualitative methods used here.**

RESPONSE: As noted above, we revised the title and aims to better reflect these points.

**It is noteworthy, for example, that treatment for anxiety involves a great deal of psychoeducation about the purpose and nature of anxiety, and then emphasizes that avoidance of things that cause anxiety lead to a worsening of anxiety. This dilemma – that people want to avoid what makes them anxious but that worsens their anxiety – is at the heart of resolving anxiety through treatment. It is worth considering how much youth, in particular, overlook this and focus only on making the anxiety go away in the moment or taking their** **attention off of it momentarily.**

RESPONSE: We appreciate this important point, and also made changes to our discussion to better describe and clarify points regarding adaptive and maladaptive avoidance in the treatment of anxiety (also see below response).

**Table 1 has some inconsistencies that need correction. Stage 2 Purpose does not really seem to be “Challenges & Pain points” though that is the text in that box. Rather, the purpose seems to be “define the problem” which is in the box defining the method of that stage.**

RESPONSE: Thank you for pointing out this inconsistency. It has been changed as recommended

**The text describing Stages 1 and 2 start with a sentence on the objective of that stage. The text describing Stage 3 should also begin this way. The description of stage 3 makes it clear that “treatments” are not the intent of that research. Identifying appropriate coping strategies and support identification is what was defined by the research process. While this is also important, it is not treatment so should not be conflated as such. The exclusion criteria ensured that depressed, self-harming and suicidal individuals were excluded. This is consistent with a research design to finding coping strategies and supports. With the less severely ill population, coping strategies and supports are appropriate. But the writing of each section, including the title and the abstract, need to reflect this so that the reader is not expecting to hear about treatment interventions.**

RESPONSE: Thank you for your thoughtful feedback and your attention to making sure that the description of our research remains focused on identifying coping strategies and supports rather than treatment interventions. In addition to addressing inconsistencies throughout the manuscript, we will address your comments in the manuscript as follows:

- **Objective for Stage 3:** we revised the paragraph describing Stage 3 to begin with a clear objective statement (similar to Stages 1 and 2) emphasizing that the focus of this stage was to explore coping strategies and support mechanisms, not treatment interventions. (p. 7)
- **Clarification of Intent**: To ensure clarity that the manuscript is focused on coping strategies and engagement rather than clinical treatment interventions, we refined the language in the introduction, stage descriptions, abstract, and title to consistently reflect this distinction (throughout).
- **Exclusion Criteria**: We reinforced in the description of exclusion criteria that the study population was not severely ill and that our research is aligned with identifying coping strategies and supports for this specific population. (p.8)

**It would be helpful to know if and to what extent there was overlap in the student-participants across the different activities (stages) of the study.**

RESPONSE: Thank you for pointing this out. We added a sentence in Data Collection/Design Thinking p.10 that indicates that all participants in the KIIs were invited to participate in the DT workshop. In results, we included that there was only one student who participated in both parts of the study. (first paragraph of page 21).

**In the Discussion the mention of technology is raised. There is acknowledgement that the youth in this study liked to use technology for “distraction from their anxiety” but evidence in the literature shows that technology fails to improve engagement and retention in treatment. These are not contradictory, since distraction and treatment are different. Again, the distinction between treatment and coping mechanisms (including distraction) are blurred in the manuscript and need to be clarified. Since the primary treatment for anxiety is reducing avoidance it is possible that it will be difficult for a technological solution to be forthcoming, though research on that topic would be timely. It is also unlikely that a young person would spontaneously recognize that reducing and eliminating avoidance is the solution to their anxiety challenges without having engaged in some actual treatment. It certainly did not come up in the conversations with youth described here.**

RESPONSE: This is a great point. Literature shows that simply putting an EBI online does not necessarily improve engagement. However, since technology helps reduce many structural barriers and is accessible for teens, we were interested in understanding if there is a way to incorporate technology to improve engagement with treatment. Importantly, our findings showed that participants used technology as a distraction from their anxiety (p.15-16) as well as suggested it would be a valuable tool for finding information about anxiety and coping strategies (p. 19-20). Further, our findings did not imply that technology alone can substitute for effective treatment. Findings showed that technology could be an effective tool for engaging in treatment if used in conjunction with other coping strategies and the involvement of a trusted adult. We revised our discussion of how youth described technology to clarify these important distinctions of adaptive and maladaptive use of avoidance/distraction techniques and better reflect these points (p. 27)

**Overall, then, this research has much to offer. Yet the description of it provided here has some inconsistencies that need to be clarified.**

**Reviewer #2:**

**The Manuscript "Using a youth-centered approach to design an intervention for the treatment of anxiety in low-income Hispanic youth" (PONE-D-24-23088) does not represent a significant contribution to the literature.**

**1. Redundancy in Target Population and Culturally Adapted Interventions
The manuscript focuses on developing a culturally relevant, youth-centered intervention for low-income Hispanic adolescents with anxiety. However, similar interventions have already been developed and published. For instance, the "Streamlined Pediatric Anxiety Program" (SPAP) has effectively designed and implemented an anxiety intervention tailored for Hispanic youth in school settings (Pina et al., 2023). SPAP utilized user-centered design and participatory research methods to adapt evidence-based interventions (EBIs) for real-world application among diverse populations, including low-income Hispanic communities.**

RESPONSE: Thank you for your comments and sharing these studies. While prior interventions like SPAP and FIRST addressed anxiety in Hispanic youth, we believe our work represents a distinct contribution by specifically engaging adolescents in a design-thinking process personalized to their lived experiences and preferences. In the references provided, Pina et.al., the redesign was for a program for elementary school students, and they were not involved in the redesign process. On page 10 of Pina et al., one of the limitations discussed is “Youth involvement in the redesign process could have made the out-of-session skill practice more attractive in ways that improve compliance. Perhaps future studies can examine if youth participatory action research, for example, yields better solutions that improve out-of-session skill practice compliance.”

Our research method emphasized co-creation with adolescents, which goes beyond adapting existing evidence-based interventions (EBIs). This emphasis on participatory design with adolescents remains underrepresented in current literature, especially for mental health interventions in Hispanic populations. We adjusted the Methods section to clarify that we used the first three phases of Design Thinking to better define the problem and that the goal of our research was not to create a full intervention but rather to explore problem domains and potential criteria for youth-centered engagement in mental health support (p.5)

**Additionally, the "Family Intervention for Resilience and Strengthening" (FIRST) program has demonstrated success in culturally tailoring anxiety interventions for Hispanic families (Weersing et al., 2017). FIRST integrates family-based support, addressing cultural nuances and family dynamics pertinent to Hispanic populations. The PLOS ONE manuscript does not offer novel cultural insights or methods beyond what these established programs have already achieved.**

RESPONSE: We reviewed Weersing publication from 2017 and were unable to find a detailed description or mention of the FIRST program. We do, however, appreciate the citation provided, which reviews depression treatment trials, of which a few included ~15-30% samples of Hispanic youth. The review noted that “…most investigations do not include large numbers of ethnic and racial minority youth, limiting power to detect effects. Comparing *across* trials, there is evidence that EBTs for depression can work well in both non-Hispanic White and diverse samples of children and adolescents (cf. [Rosselló & Bernal, 1999](https://pmc.ncbi.nlm.nih.gov/articles/PMC5296370/#R108); [Weisz et al., 1997](https://pmc.ncbi.nlm.nih.gov/articles/PMC5296370/#R139)), but direct tests of this association *within* trials are lacking.” Although focused on depression, this aligns with our argument and premise that Hispanic youth are not sufficiently being engaged in effective mental health treatment research. Hence, our study aimed to explore ways to improve engagement by involving Hispanic youth more directly in the design process.

**2. Established Digital and Technology-Based Intervention Models**
**The manuscript proposes a technology-based program to engage Hispanic adolescents in managing anxiety. However, this approach is not novel, as existing programs have successfully integrated digital tools into anxiety interventions. The SPAP incorporates gamification and digital resources to enhance engagement and accessibility for youth (Pina et al., 2023). Similarly, digital interventions targeting adolescent anxiety have been explored extensively, with studies demonstrating their effectiveness in increasing engagement and reducing symptoms (Hollis et al., 2017; Wozney et al., 2018). Therefore, the manuscript's digital approach does not constitute a significant advancement over existing literature**.

RESPONSE: Thank you for your feedback. We acknowledge that digital interventions like SPAP and those discussed by Hollis et al. (2017) and Wozney et al. (2018) have demonstrated effectiveness in addressing adolescent anxiety, however, our study differs in that it does not propose a specific solution. Instead, the goal of our study was to identify key domains that are crucial for improving engagement with mental health interventions and to inform future design. Our study also emphasizes how adolescents already use technology (e.g., social media, games) and explores how this can inform the development of more engaging, culturally responsive interventions. Our research methods focus on understanding adolescent preferences and how these can be incorporated into the design process to improve engagement and usability. This is particularly important for Hispanic youth, who often face barriers to traditional mental health services. Our findings did not imply that technology alone can substitute for effective treatment. Findings showed that technology could be an effective tool for engaging in treatment if used in conjunction with other coping strategies and the involvement of a trusted adult.

By emphasizing adolescent involvement from the beginning, our goal is to ensure that interventions are more relevant, culturally responsive, and effective in engaging this population. These insights are intended to guide the development of future programs that will better meet the needs of youth facing anxiety.

To help clarify this, we made modifications to the manuscript that emphasize design for engagement rather than for a complete intervention.

**3. Comprehensive Addressing of Psychosocial Barriers**
**The identification of psychosocial and structural barriers to mental health service utilization among Hispanic youth is well-documented. Both the SPAP and FIRST programs have addressed these barriers by incorporating strategies to reduce stigma, enhance trust, and improve accessibility (Pina et al., 2023; Weersing et al., 2017). The manuscript under review reiterates these known barriers without providing new strategies or insights to overcome them, resulting in a lack of significant contribution to the field.**

RESPONSE: We agree that psychosocial barriers are well-documented, however, our research offers new insights by engaging youth directly to discover differences in how these barriers impact their specific engagement with mental health services. Using a youth-centered approach allowed us to identify innovative, personalized strategies, specific to Hispanic cultural contexts and the developmental aspects unique to adolescence. This method deepens our understanding of how adolescent identity and agency interact with known psychosocial challenges, which has implications for improving intervention effectiveness and adherence.

**4. Lack of Theoretical Innovation**
**The manuscript relies on Social Cognitive Theory and participatory research methods**, **which have been extensively applied in prior studies. The SPAP, for example, used a "small theory" approach integrating cognitive-behavioral strategies and user-centered design (Pina et al., 2023). The FIRST program also employs evidence-based theoretical frameworks in its intervention design (Weersing et al., 2017). The manuscript does not offer novel theoretical perspectives or methodological advancements beyond these established frameworks.**
RESPONSE: Our work applies Social Cognitive Theory in a unique way within a design-thinking framework that is focused on adolescent agency and engagement. This approach integrates cognitive-behavioral principles with iterative design phases where adolescents have an active role in providing theoretical and methodological innovation. The integration of theory with a youth-centered design process is new because it empowers adolescents to contribute directly to intervention content and delivery mechanisms which differentiates our study from others that primarily adapt interventions without this level of adolescent involvement.

**5. Proven Efficacy of Existing Interventions**
**Existing interventions like SPAP and FIRST have demonstrated efficacy through rigorous evaluations, including randomized controlled trials. SPAP showed significant improvements in self-efficacy, reductions in anxiety symptoms, and high levels of participant engagement (Pina et al., 2023). FIRST has similarly shown positive outcomes in anxiety reduction among diverse youth populations (Weersing et al., 2017). The PLOS ONE manuscript does not provide evidence that its proposed intervention would yield superior or additional benefits compared to these established programs.**

RESPONSE: We appreciate that effective treatments exist; however, as we note in our introduction, youth with mental health needs are not engaging in these treatments, or when they do, adherence and retention remains challenging. As such, our study does not propose an intervention but rather focuses on working with youth, the persons seeking treatment, to define the problem and offer possible solutions to increase engagement with treatment. This addresses a gap in the existing literature by focusing on factors that impact adolescent engagement, retention, and usability of an intervention which are critical outcomes for the development of effective interventions. By involving adolescents in the design process, we aim to improve adherence and long-term effectiveness, which are challenging with traditional interventions. Our research seeks to enhance the depth of engagement specifically within the adolescent Hispanic population. Our findings indicate unique adolescent-driven elements that may lead to higher engagement than previously reported in other studies.

**6. Redundancy in Identified Themes and Strategies**
**The themes identified in the manuscript—such as barriers to seeking help, preferred coping strategies, and the importance of culturally relevant interventions—have been extensively explored in existing literature. The SPAP addresses these themes by integrating them into a streamlined, practical program suitable for school settings (Pina et al., 2023). Reiterating these established findings without adding new knowledge diminishes the manuscript's contribution.**

RESPONSE: We agree that themes of accessibility and cultural relevance are widely known, however, we believe this study introduces a fresh perspective by examining these themes from an adolescent perspective where technology is widely used for distraction from anxiety and for researching the condition and effective coping strategies. The iterative design process used in Design Thinking allowed adolescents to express these themes as they relate to their specific experiences, which can reveal new insights that are not captured in traditional research approaches. For example, in our study, youth indicated ways in which music could be a coping strategy as well as a useful engagement tool by developing and sharing special playlists of their favorite songs and artists related to how they are feeling. This level of participant involvement contributes additional knowledge on adapting mental health interventions to fit the unique cultural and developmental needs of adolescents.
